# Association of gestational weight gain with cesarean section: a prospective birth cohort study in Southwest China

**DOI:** 10.1186/s12884-020-03527-1

**Published:** 2021-01-14

**Authors:** Lujiao Huang, Ju Zhang, Hong Sun, Hongli Dong, Run Li, Congjie Cai, Yan Gao, Cheng Wu, Xi Lan, Guo Zeng

**Affiliations:** 1 West China School of Public Health and West China Fourth Hospital, Chengdu, China; 2 Department of Clinical Nutrition, Sichuan Provincial Hospital for Women and Children, Chengdu, China; 3 Department of Clinical Nutrition, Chengdu Fifth People’s Hospital, Chengdu, China

**Keywords:** Gestational gain weight, Cesarean section, Optimal recommendation, Birth cohort study, Chinese population

## Abstract

**Background:**

Cesarean section (CS) is a rising public health issue globally, and is particularly serious in China. Numerous studies have suggested that gestational weight gain (GWG) control may be an effective way to reduce the rate of CS. However, rare study has examined the association between GWG and CS among women in Southwest China. We proposed to examine their association based on a prospective birth cohort, and further to explore the optimal GWG range.

**Methods:**

We retrieved data from a prospective birth cohort from Sichuan Provincial Hospital for Women and Children, Southwest China. Unconditional multivariable logistic regression was used to examine the association between GWG and CS by adjusting for potential confounders. In one analysis, we incorporated the GWG as a categorical variable according to the Institute of Medicine (IOM) recommendation, similar to the method used in the majority of previous studies. In the other analysis, we directly incorporated GWG as a continuous variable and natural cubic splines were used to characterize the potential nonlinear exposure-response relationship, aiming to identify the optimal GWG. We further stratified the above analysis by pre-pregnancy BMI and GDM, and then a heterogeneity test based on a multivariate meta-analysis was conducted to examine whether the stratum specific estimations agreed with each other.

**Results:**

A total of 1363 participants were included. By adopting the IOM recommendation, the adjusted OR of CS was 0.63 (0.47, 0.84) for insufficient GWG and 1.42 (1.06, 1.88) for excessive GWG. After stratification by pre-pregnancy BMI, we found a higher risk of CS in associated with excessive GWG in the stratum of underweight compared with the other strata, which implied that pre-pregnancy BMI may be an effect modifier. By applying a flexible spline regression, the optimal GWG levels in terms of reducing the CS rate based on our data were more stringent than those of IOM recommendation, which were 9–12 kg for underweight women, < 19 kg for normal weight women and < 10 kg for overweight/obese women.

**Conclusions:**

These results suggested that a more stringent recommendation should be applied in Southwest China, and that more attention should be given to underweight women.

## Background

Over the past few decades, a rising rate of cesarean section (CS) has been seen globally [1]. In China, it has increased dramatically from 3.4% in 1988 to 39.3% in 2008 [2]. Although the overall use of CS in China has stabilized at approximately 40–50% in recent years [3], these rates are still three times higher than the ideal CS rate (i.e. 10 ~ 15%), and greatly exceed the warning line of 15% recommended by the World Health Organization [4]. In addition, the CS rate without medical indications in China was 11.6%, which was also much higher than that in Western developed countries and other Asian countries [5]. In Southwest China, the situation is even worse. Taking Chengdu city (a supercity in Southwest China with over 16 million people) as an example, its CS rate reached an astonishing 57.0% in 2014, which was far higher than the average rate in China [6].

It is well acknowledged that CS without medical indications may be associated with multiple adverse maternal and infant outcomes [7]. For pregnant women, CS has been associated with an increased risk of hemorrhage, embolism, intra-abdominal adhesions, and even death [8]. For the offspring, CS has been associated with an increased risk of developing asthma, allergies, cardiometabolic syndromes, and poor psychological health later in life [9–11]. Therefore, preventing the overuse of CS has become an urgent need for maternal health in China. An effective way to address the above issue is to identify the modifiable risk factors and to formulate the corresponding intervention to reduce the rate of CS.

Numerous risk factors, such as gestational weight gain (GWG), pre-pregnancy body mass index (BMI), and gestational diabetes mellitus (GDM), have been suggested to be associated with CS [12, 13]. Among them, GWG has aroused considerable attention owing to its potential to be an intervention target. However, most of the previous evidence regarding the association between GWG and CS came from Western developed countries [14]. Given that the BMI of pre-pregnancy women in China is substantially lower than that in western developed countries, those findings may not be generalizable to the Chinese population. In addition, the majority of previous studies in China and other Asian countries used the guideline from the Institute of Medicine (IOM, currently the US national academy of medicine) to classify the GWG into different groups and to study its association with CS [12, 15–17]. Similar to the above, it is also questionable whether the IOM guidelines can be the optimal recommendation to reduce the CS rate in China.

To fill the above research gaps, we proposed to study the association between GWG and CS based on a prospective birth cohort from Southwest China. Unlike most previous studies that adopted the IOM guideline to classify the GWG, we additionally used a flexible spline regression to characterize the potential nonlinear exposure-response curve between GWG and CS, aiming to identify the optimal recommendation of GWG in Southwest China to reduce the CS rate.

## Methods

### Study population

**Fig. 1:**
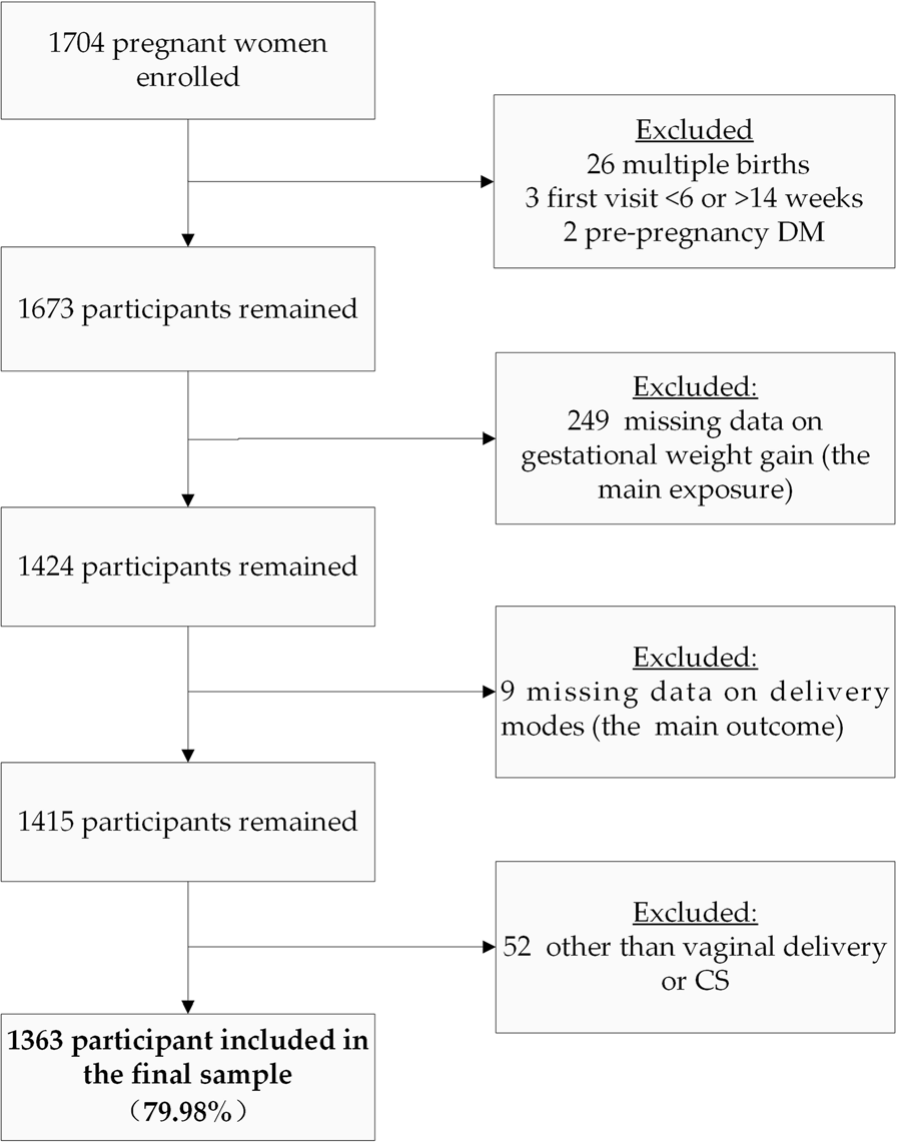
The flowchart of the analysis sample selection

Data used in this study were retrieved from a prospective birth cohort in Sichuan Provincial Hospital for Women and Children, Southwest China. Data collection was approved by the Institutional Review Board at Sichuan University (approval number: K2017037). More details about this birth cohort and the questionnaire used in this study have been described elsewhere [18]. In summary, pregnant women who had their first prenatal clinic visit were invited to join this birth cohort in 2017. Eventually, 1704 pregnant women were recruited and followed up until two years after delivery. Among these women, 26 were excluded for multiple births, 3 were excluded for first prenatal clinic visit < 6 or > 14 weeks, 2 were excluded for pre-pregnancy diabetes mellitus, and 249 were excluded for having missing data on the main exposure variable (i.e., gestational weight gain), and 9 were excluded for having missing data on the main outcome variable (i.e., delivery modes). In addition, 52 participants were further excluded due to delivery modes other than vaginal delivery or cesarean section. Finally, 1363 participants with maternal age ranging from 18 to 45 years old were included in the final sample for the statistical analysis (Fig. 1).

### Data collection and anthropometric measurements

After enrollment and providing written informed consent, all participants were required to complete sequential interviewer-administered questionnaires given by trained staff at different routine prenatal and child health visits. The baseline questionnaire covered a wide range of information about the pregnant mother, including general demographic and socioeconomic characteristics, lifestyle habits (e.g. pre-pregnancy drinking and smoking habits), physical activity, dietary, mental and sleeping status quality, reproductive history and etc. Specifically, physical activity was assessed by the pregnancy physical activity questionnaire (PPAQ) [19], which investigated the activity of participants in self-care, housework, leisure activity, transportation and work. Dietary was evaluated through a 24-hour dietary recall survey. Depression and anxiety were evaluated by the Self-Rating Anxiety Scale (SAS) and the Self-Rating Depression Scale (SDS) respectively. The sleeping quality was evaluated by the Pittsburgh sleep quality index scale (PSQI). In particular, the physical activity, and dietary, mental and sleeping status quality were evaluated three times in the first, second and third trimesters. After childbirth, the maternal outcomes were recorded by use of a standardized form including the delivery mode, maternal weight and glucose after delivery, breastfeeding initiation, etc.

Regarding the anthropometric measurements, we only measured height in meter once at enrollment by a standardized height-measuring station. However, we measured prenatal weight in kilograms four times from the first to third trimesters and before delivery using a standardized weight scale. For the pre-pregnancy weight, we used the self-reported data by directly asking the participants at enrollment. We also retrieved the 75-g OGTT results at 24–28 weeks of gestation from the hospital database to further determine whether the participant experienced gestational diabetes mellitus (GDM). GDM was diagnosed according to the standards issued by the Ministry of Health of China in 2011, which were based on the International Association of Diabetic Pregnancy Study Group guidelines [20]. In summary, GDM was defined as having one or more abnormal values: 5.1 mmol/l or 92 mg/dl for fasting, 10.0 mmol/l or 180 mg/dl for 1-h post-load and 8.5 mmol/l or 153 mg/dl for 2-h post-load glucose.

### Assessment of study variables

The GWG in this study was defined by the difference between the last measured weight (i.e., prenatal weight before delivery) and the pre-pregnancy weight. Then we classified the GWG of each participant into three groups (i.e., below, within and above) according to the IOM recommendations [21], but with the pre-pregnancy BMI stratified by the Chinese recommendation: underweight (BMI < 18.5 kg/m^2^), normal weight (18.5 ≤ BMI < 24.0 kg/m^2^), overweight (24.0 ≤ BMI < 28.0 kg/m^2^) and obese (BMI ≥ 28.0 kg/m^2^). In the final analysis, we further combined the overweight and obese groups due to their being only a few participants in the obese group. For the outcome variable, we classified the delivery modes into two types in our questionnaire, i.e., vaginal delivery and CS.

A wide range of potential covariates were available for careful consideration of confounding factors. The definitions of those covariates are displayed as follows. They included maternal age in years (< 25, 25–29, 30–35, ≥ 35), ethnicity (Han, minorities), employment before pregnancy (yes, no), personal income per month in Chinese Yuan(< 3000, 3000–4999, 5000–9999, ≥ 10,000), education in schooling years (≤ 12 years, 13–15 years, ≥ 16 years), drinking status before pregnancy (yes or no), smoking status before pregnancy (yes or no), gravidity (1, 2, 3, ≥ 4), parity (primiparous or multiparous), physical activity (MET-h/week), energy intake (kcal/day), anxiety status measured by the SAS score, depression status measured by the SDS score and sleep quality measured by the PSQI score.

### Statistical analysis

Unconditional multivariable logistic regression was used to examine the association between GWG and CS (vaginal delivery as reference) by adjusting for the following confounding variables: maternal age, gravidity, parity, ethnicity, educational level, employment, personal income, smoking, drinking, physical activity, energy intake, anxiety, depression, and sleeping status. The determination of potential confounders was informed by a thorough review of previous studies and by consulting expert opinions. We conducted two separate analyses with different definitions of the main exposure (i.e., GWG) for different purposes. In one analysis, we incorporated the GWG as a categorical variable according to the IOM recommendation, similar to the majority of previous studies. In the other analysis, we directly incorporated the GWG as a continuous variable and natural cubic splines were used to characterize the potential nonlinear exposure-response relationship. In addition, we ran a threshold effect analysis based on segmented regression to identify the threshold/boundary value [22], aiming to identify the optimal GWG in terms of reducing the CS rate in our study population. We further stratified the above analysis by pre-pregnancy BMI and GDM considering that our conclusions could be profoundly modified by those two factors. Then a heterogeneity test based on multivariate meta-analysis was conducted to examine whether the stratum specific estimation agreed with each other. All statistical analyses in this study were conducted in R software version 4.0.2.

## Results

A total of 1363 participants were included in this analysis from the Prospective Birth Cohort Study in Southwest China, with pre-pregnancy overweight/obese, excessive GWG and CS rates of 12.7%, 27.6% and 43.7% respectively. We compared the sample characteristics between the vaginal delivery and cesarean section groups in Table 1. Compared to the vaginal delivery group, participants in the CS group generally had a higher rate of excessive GWG (33.0% vs. 23.4%, *P* < 0.001) as well as overweight/obese (17.1% vs. 9.3%, *P* < 0.001). However, the rate of gestational diabetes mellitus between these two groups showed no significant difference (39.4% vs. 36.7%, *P* < 0.001). In addition, we also found that the maternal age ≥ 35 (13.6% vs. 5.6%, *P* < 0.001), gravidity ≥ 3 (35.1% vs. 20.5%, *P* < 0.001) and multiparous rate (38.5% vs. 23.5%, *P* < 0.001) of the CS group were higher than those of vaginal delivery group, while no significant differences were found for any other characteristics between the two groups.

**Table 1:** Sample characteristics between vaginal delivery and cesarean section

Characteristics^a^	Overall (*n* = 1363)	Vaginal delivery (*n* = 767, 56.3%)	Cesarean section (*n* = 596, 43.7%)	*χ*^*2*^/T^*^	*P*
GWG (continuous, kg)	13.5 (10.5, 16.2)	13.1 (10.5, 16.0)	13.9 (11.0, 16.8)	8.072	0.004
GWG (categorical, kg)					
Below	382 (28.1)	245 (32.0)	137 (23.0)	21.102	< 0.001
Within	604 (44.3)	342 (44.6)	262 (44.0)		
Above	376 (27.6)	179 (23.4)	197 (33.0)		
Pre-pregnancy BMI (kg/m^2^)					
Under weight (< 18.5)	195 (14.3)	132 (17.2)	63 (10.6)	26.697	< 0.001
Normal weight (18.5 ~ 24)	994 (73.0)	563 (73.5)	431 (72.3)		
Overweight/obese (≥ 24.0)	173 (12.7)	71 (9.3)	102 (17.1)		
Gestational Diabetes Mellitus					
Yes	515 (37.8)	281 (36.7)	234 (39.4)	0.932	0.334
No	848 (62.2)	486 (63.3)	362 (60.6)		
Maternal age (years)					
<25	232 (17.1)	151 (19.7)	81 (13.6)	47.035	< 0.001
25–29	716 (52.5)	436 (56.8)	280 (47.0)		
30–35	291 (21.3)	137 (17.9)	154 (25.8)		
≥ 35	124 (9.1)	43 (5.6)	81 (13.6)		
Ethnicity					
Han	1334 (97.9)	755 (98.6)	579 (97.1)	2.673	0.102
Minorities	29 (2.1)	12 (1.4)	17 (2.9)		
Education (schooling years)					
≤ 12	314 (23.1)	165 (21.6)	149 (25.1)	2.909	0.233
13–15	954 (70.3)	545 (71.2)	409 (69.0)		
≥ 16	90 (6.6)	55 (7.2)	35 (5.9)		
Employment					
Yes	1141 (83.7)	656 (85.5)	485 (81.5)	3.685	0.055
No	222 (16.3)	111 (14.5)	111 (18.5)		
Personal income (Yuan/month)					
< 3000	46 (3.4)	24 (3.1)	22 (3.7)	1.210	0.751
3000–4999	397 (29.3)	224 (29.4)	173 (29.1)		
5000–9999	617 (45.5)	354 (46.5)	263 (44.3)		
≥ 10,000	296 (21.8)	160 (21.0)	136 (22.9)		
Gravidity					
1	594 (43.6)	377 (49.2)	217 (36.5)	39.522	< 0.001
2	401 (29.5)	232 (30.3)	169 (28.4)		
≥ 3	366 (26.9)	157 (20.5)	209 (35.1)		
Parity					
primiparous	942 (70.0)	580 (76.5)	362 (61.5)	35.025	< 0.001
multiparous	405 (30.0)	178 (23.5)	227 (38.5)		
Drinking status					
Yes	107 (29.5)	59 (7.7)	48 (8.1)	0.021	0.883
No	1256 (70.5)	708 (92.3)	548 (91.9)		
Smoking status					
Yes	51 (3.7)	26 (3.4)	25 (4.2)	0.402	0.526
No	1312 (96.3)	741 (96.6)	571 (95.8)		
Physical activity (MET hours/weeks)	102.6 (78.9, 124.1)	101.9 (78.3, 124.4)	103.8 (80.4, 123.7)	0.381	0.537
Energy intake(kcal/day)	1724 (1496, 1976)	1734 (1509, 1994)	1702 (1455, 1968)	2.022	0.155
Anxiety (SAS score)	36.3 (32.5, 40.4)	36 (33, 40)	36 (33, 40)	0.217	0.642
Depression (SDS score)	38.8 (33.8, 44.2)	39 (33, 45)	38 (34, 44)	0.071	0.791
Sleeping (PSQI score)	4.0 (3.0, 5.0)	4 (3, 5)	4 (3, 5)	0.014	0.907

^a^The data are presented as medians and quartiles for continuous variables (all continuous variables are non-normally distributed) or as n and % for categorical variables

* Tests for differences between vaginal delivery and cesarean section were performed using the Wilcoxon two-sample test for non-normally distributed continuous variables or the ***χ***^2^ test for categorical variables; *P* < 0.05 indicates significance

For the categorical GWG, we found a consistent and steady increase in the risk of CS from below to above the IOM recommendation, as seen in Table 2. Overall, compared to those participants within the IOM recommendation, the estimated risk of CS for those below the recommendation decreased 0.63 times (95% CI: 0.47-0.84), and for those above the recommendation, the risk of CS increased 1.42 times (95% CI: 1.06-1.88). Further stratifying the analysis by BMI, although similar upward trends were found in all strata, the estimated effect size varied substantially depending on the stratum (P for heterogeneity <0.001). Particularly, we found that the underweight participants were most sensitive to excessive GWG, given that the greatest OR (2.67, 95% CI: 0.98, 7.26) was seen in the underweight stratum and above the recommendation. Our findings also implied that the overweight/obese participant would gain the most benefit by controlling their GWG under the IOM recommendation, given that the smallest OR (0.29, 95% CI: 0.09, 0.89) was seen in the overweight/obese stratum and below the recommendation. In contrast, the estimated effect size was more similar between the two strata of GDM than that of BMI (P for heterogeneity =0.010).

**Table 2:** Associations of gestational weight gain categorized by the IOM recommendation with cesarean section (vaginal delivery as a reference)^a^

IOM recommendation	Overall	Stratified by pre-pregnancy BMI				Stratified by GDM		
		**Under**	**Normal**	**Over**	${\varvec{\chi }}^{2}$**/P**^**#**^	**GDM**	**Non-GDM**	${\varvec{\chi }}^{2}$**/P**^**#**^
Below	0.63 (0.47, 0.84)	0.84 (0.36, 1.99)	0.64 (0.46, 0.89)	0.29 (0.09, 0.89)	128.5, < 0.001	0.60 (0.39, 0.95)	0.65 (0.43, 0.98)	11.5, 0.010
Within	1.00 (ref)	1.00 (ref)	1.00 (ref)	1.00 (ref)		1.00 (ref)	1.00 (ref)	
Above	1.42 (1.06, 1.88)	2.67 (0.98, 7.26)	1.36 (0.98, 1.90)	1.69 (0.66, 4.31)		1.10 (0.63, 1.94)	1.58 (1.12, 2.22)	

^a^The associations of GWG with CS were measured by the adjusted odds ratio (OR) and estimated by nonconditional multivariate logistic regression by controlling for maternal age, gravidity, parity, ethnicity, educational level, employment, personal income, smoking, drinking, physical activity, energy intake, anxiety, depression, sleeping status, pre-pregnancy BMI and GDM. For the stratified analysis, BMI and GMD were then excluded from the confounding set accordingly

^#^We ran a heterogeneity test based on multivariate meta-analysis to test whether the estimated OR in different strata differed significantly, or whether the observed differences could be attributable to random variation

**Fig. 2:**
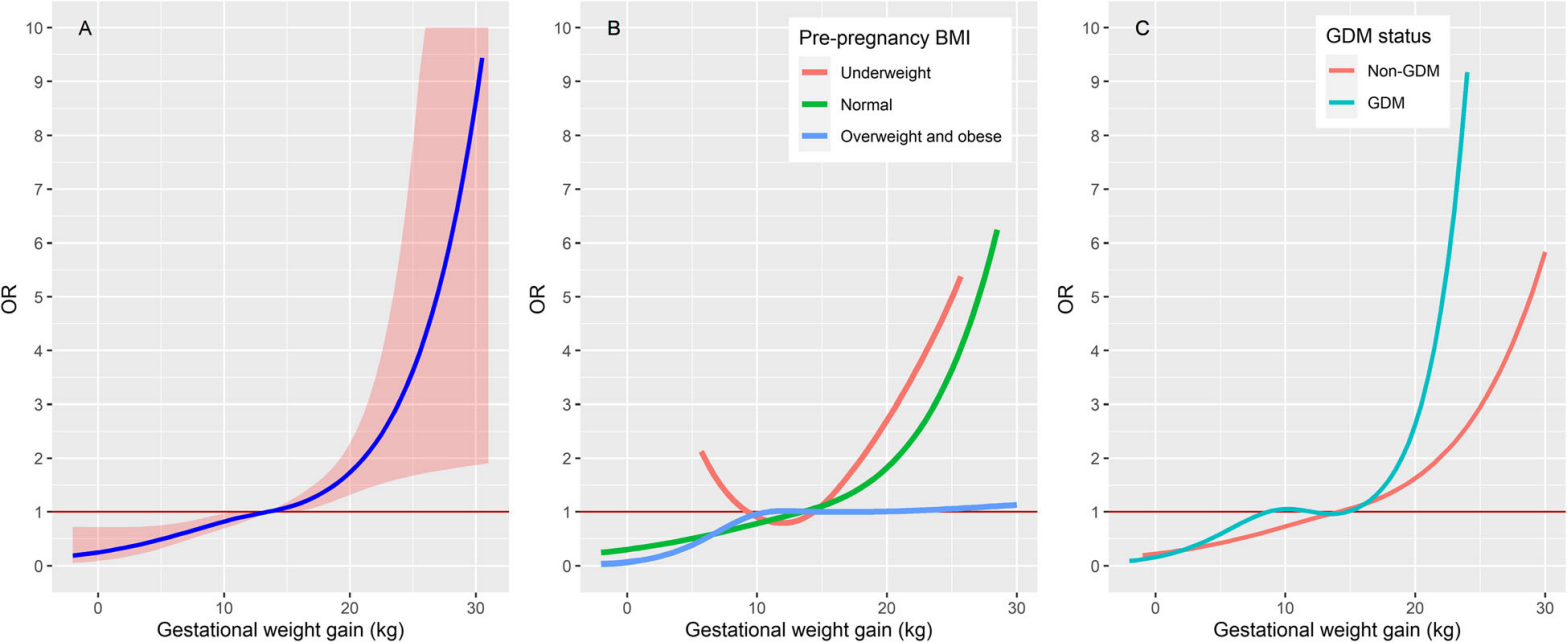
The estimated nonlinear exposure-response curve between the gestational weight gain and cesarean section. **a**: The overall estimation (95% CI as the shaded area). **b**: Estimations stratified by BMI. **c**: Estimations stratified by GDM. We did not display the 95% CI in subfigure **b** and **c** to avoid the overlapping shaded area hindering a clear comparison among the different strata

For continuous GWG, our findings generally agreed with those of categorical GWG but with more details. Overall, we found a J-shaped exposure-response curve between GWG and CS (Fig 2a). More specifically, we observed an approximately linear increase in the risk of CS until the GWG reached 19 kg. Since then, the risk of CS began to increase exponentially which implied a threshold effect. Similar to the categorical analysis, the estimated exposure-response curves varied substantially among the different BMI strata. Compared to the overall estimation, we found a similar J-shaped exposure-response curve and a threshold value of 19 kg in the normal weight stratum. However, in the underweight stratum, the estimated exposure-response curve showed a U shape. This implied that insufficient GWG would not decrease but would instead increase the risk of CS and the optimal GWG was approximately 9-12 kg. In contrast, we observed a steep increase in the risk of CS from 0-10 kg GWG for overweight or obese participants, afterward, the risk of CS began to level off. This implied that the risk of CS was very sensitive to the increase in GWG, but an excessive GWG over 10 kg would not further increase the risk of CS. Once again, we found that the difference in estimations between the GDM and non-GDM strata was much smaller than that among the BMI strata. For both groups, the estimated exposure-response curves were showed a J shape, similar to the overall estimation.

## Discussion

In this study, we examined the association between GWG and CS based on a prospective birth cohort from Southwest China. The rates of overweight/obese and excessive GWG (IOM guideline) in our study were 12.7% and 27.6% respectively. Our findings agreed with previously reported large scale national investigation data in China (12.7% and 27.6%) [15, 23]. However, both rates were significantly lower than those in Western countries (25.8% and 47.2%) [14, 24], and also the neighbouring Asian countries, such as Japan (20.8% and 37.2%) [25] and South Korea (20.5% and 35.6%) [26]. Our findings confirmed that the women in Southwest China were slimmer than those in developed counties. Not surprisingly, we found a high rate of CS in Southwest China (43.7%). This finding was consistent with the national survey of CS conducted by the National Maternal Near Miss Surveillance System (41.1%) [3]. Although there is an increasing trend in CS globally, the rate of CS in Southwest China is still much higher than that in most other countries, ranging from 7.3% in Africa to 32.3% in North America [27]. The only exceptions were Latin America and the Caribbean region where the rate of CS reached 40.5% [27].

Previous studies have shown that both pre-pregnancy BMI and GWG were associated with the risk of CS [12, 14, 17]. In this study, we paid particular attention to the GWG rather than the pre-pregnancy BMI, mainly because it is more feasible for weight control during the gestation period than before pregnancy [12]. By adjusting for potential confounding factors as well as pre-pregnancy BMI, we found a steady increase in the risk of CS with increasing GWG with estimated ORs for the insufficient and excessive GWG of 0.63 (0.47, 0.84) and 1.42 (1.06, 1.88), respectively. Our results were roughly consistent with most previous studies. Taking the risk of CS in the setting of excessive GWG for example, the estimated ORs were 1.44 (1.21–1.72) in the United States [28], 1.45 (1.40, 1.51) and 1.44 (1.20,1.73) in mainland China [12, 15], 1.35 (1.16–1.56) in Chinese Taiwan [29], 1.36 (1.25, 1.47) in Japan [25], 1.6 (1.0–2.7) in South Korea [30] and 1.9 (1.4–2.5) in India [31]. Several possible reasons could explain the association between excessive GWG and CS. Excessive GWG may increase the risk of multiple adverse maternal and infant outcomes [32, 33], such as poor performance on the Apgar score, macrosomia and foetal distress, and dysfunction in myometrial contractility of the pregnancy mothers, thereby increasing the risk of cesarean delivery.

In addition, our results implied that the pre-pregnancy BMI may modify the association between GWG and CS. After stratifying our analysis by BMI, the most increased risk of CS due to excessive GWG was seen in underweight women, while the most reduced risk of CS due to insufficient GWG was seen in overweight women. These findings were consistent with those of previous studies conducted in both China [12] and the USA [34]. The findings of those studies implied that excessive GWG may do more harm for underweight women than their obese counterparts regarding the risk of CS. In most clinical practices, weight control is mainly recommended for overweight women. However, our results also suggested that we should pay more attention to the issue of excessive GWG for underweight women. In contrast, the modification effect of GDM on the GWG-CS association was negligible.

In recent years, an increasing number of studies have begun to question the IOM guidelines for GWG for two main reasons. One is that the IOM guideline is developed mainly based on the Caucasian standard, thus it may not be applicable to other racial and ethnic groups [35]. Another is that the IOM guideline has limitations in providing insufficient information on adverse outcomes (only 5 maternal and offspring outcomes were considered in developing the IOM guideline), thus it may not be applicable to other adverse outcomes [14]. To explore the optimal GWG in terms of reducing CS in the Chinese population, we additionally incorporated the GWG as a continuous variable into a flexible spline regression model. For normal weight women, we found that the risk of CS increased exponentially once the GWG exceeded 19 kg, which implied a threshold effect and the optimal GWG should be controlled under 19 kg. For those underweight and overweight/obese women, the recommendations based on our findings were 9–12 kg and < 10 kg respectively. Compared to the IOM guidelines [21], our recommendation was more stringent and lower than that of IOM, especially for underweight women. This finding was generally consistent with that reported in Japan [36] and Singapore [37]. However, the results from South Korea [35] suggested a considerably higher and wider optimal GWG range than the IOM guidelines.

To the best of our knowledge, this is the first study in southwest China aiming to examine the association between GWG and CS. Compared with other studies, we have taken a large number of covariates into account to allow for stringent control of potential confounding factors. In addition, the sophisticated natural spline regression adopted in this study can also allow us to achieve a fine characterization of the association between GWG and CS, thereby exploring the optimal GWG ranges. However, this study also has several limitations that warrant mentioning. The first is that the sample size of this analysis is relatively small, which may hinder the representativeness and generality of this study. For this reason, we did not distinguish elective and non-elective cesarean sections in our analysis to avoid insufficient statistical power. However, for the selective CS, their choice of CS may have nothing to do with the GWG, thereby attenuating our estimates of the association between GWG and CS. The second is that we did not take the change in GWG over different trimesters into account in our analysis. The main reason we did not use the trimester-specific GWG is that the rate of missing data would be much higher than that when using the overall GWG. Therefore, we chose the overall GWG as our main exposure to obtain a larger effective sample size. The third has to do with the SDS used in this article to measure depression during pregnancy. According to a recent study, the SDS needs modification before being applied to pregnant women in China based on a discrimination and structural validity evaluation [38]. This may have an impact on our findings. However, the modification of the SDS was far beyond the scope of this study. The fourth is that we found that those included participants had a slightly higher level of maternal age, education level, and energy intake, and slightly lower level of anxiety and depression, compared to those not included. Therefore, we cannot rule out the possibility of that our findings may be affected by the potential selection bias. Finally, we did not collect the needed information to exclude repeated CS. However, the proportion of repeated CS is usually very low in China, so it should not have a substantial impact on our results.

## Conclusions

Overall, we investigated the association between GWG and CS in a population from Southwest China based on a prospective birth cohort. We confirmed that an elevated risk of CS was associated with an increase in GWG in accordance with the IOM recommendation. Our findings also suggested that the risks may vary by the pre-pregnancy BMI. Particularly we should pay more attention to an excessive GWG for underweight women because this may increase the risk of CS compared to others. Overall, our results also suggested that a more stringent recommendation of GWG should be applied in Southwest China.

## Data Availability

The datasets analyzed in this study are available from the corresponding author on reasonable request.

## Abbreviations

GWG: Gestational gain weight
CS: Cesarean section
IOM: Institute of Medicine
OR: Odds ratio
BMI: Body mass index
GDM: Gestational diabetes mellitus
SAS: Self-Rating Anxiety Scale
SDS: Self-Rating Depression Scale
PSQI: Pittsburgh sleep quality index scale
